# Comparison of augmented reality glasses for the assistive communication support of hearing loss

**DOI:** 10.3389/fneur.2025.1635699

**Published:** 2025-11-05

**Authors:** Helge Rhodin, Imran Ersoy, Sefa Aygun, Christoph J. Pfeiffer, Anna Lisa Vollmer, Ingo Todt

**Affiliations:** ^1^Faculty of Technology, AG Visual AI for Extended Reality, Bielefeld University, Bielefeld, Germany; ^2^Department of Otolaryngology, Medical Faculty OWL, Bielefeld University, Campus Klinikum Bielefeld Mitte, Bielefeld, Germany; ^3^AG Interactive Robotics, Medical Faculty OWL, Bielefeld University, Bielefeld, Germany

**Keywords:** augmented reality, artificial intelligence, hearing loss, indications of sign language, speech to text transcripts

## Abstract

**Background:**

Augmented reality (AR) glasses can be utilized for various medical applications. Primarily, a visual overlay on the optic screen offers additional operational information. A transfer of acoustic information via speech-to-text transcript using AR glasses presents a new non-surgical option to support patients with different forms of hearing loss. This study aimed to evaluate different AR glasses for speech-to-text transcription.

**Methods:**

We compared four different AR glasses systems (Even Realities, G1; Meizu, MYVU IMIKI; XREAL, AIR, and Epson, Moverio 40) in terms of speech-to-text transcription, design, software, microphone and connection in this laboratory based study. Speech-capturing ability was tested using free-field numbers, monosyllables, and OLSA in quiet and in noise.

**Results:**

The AR systems achieved Freiburger monosyllabic speech recognition rates between 20 and 45% at 65 dB. OLSA in quiet results vary between 77 and 100%, with increases of +1.7 dB and +3.5 dB in noise. AR systems differ substantially in terms of design, software, microphone position, and connection. Proposed indication groups are given.

**Conclusion:**

AR glasses provide a potential supportive tool for patients with specific indications suffering from hearing loss. The systems show limitations in challenging hearing situations.

## Introduction

The gaming industry, as a key driver for the introduction of extended reality (XR), played a central role in the distribution of augmented reality (AR) and virtual reality (VR) glasses. While VR sets the user in a complete digital visual environment, AR provides a visual overlay on top of real-world perception. Visual capture and overlay by AR and VR are expected to revolutionize how we interact with both the digital and real worlds (1).

Although they have been used so far primarily in non-regular daily settings, various applications, even in otolaryngology, have been tested and described. In a cadaveric study, Chan et al. utilized AR with TORS (trans oral robotic surgery) to enhance the identification of critical structures. Other groups have attempted to utilize AR to enhance parotid surgeries relevant to structure identification (2). Further, it was used for free flap harvesting and reconstructive planning (3).

Otology can be assumed to be a primary driver in digital visual applications. By definition, the ROBOSCOPE system (BHS, Innsbruck, Austria) is a VR system that enables high-precision surgery, such as cochlear implantation, free flap anastomosis, and neurosurgical procedures (4, 5). Even here, augmenting electrophysiological information is helpful (6) and can be implemented into the surgical procedure.

AR-guided surgery has been used to implant bone-conductive devices, improving accuracy (7). An AR system guiding through the Da Vinci Si System was used to perform cadaveric mastoidectomies, posterior tympanotomies, and cochleostomies (8).

However, AR has much more to offer than just an overlay of additional, primarily anatomic visual information. Cross-modal sensory interaction is well-established and applied in various clinical fields, such as tinnitus and vestibular rehabilitation. The principle of the Lenire system involves a tongue stimulator designed to alleviate the burden of tinnitus (9). This concept is based on the approach described by Danilov et al. (10), where a device called the “BrainPort” stimulates the tongue to enhance balance control. In this context, the principle is applied when head movements deviate from an acceptable range, helping to improve balance.

A mixed-case example is the successful use of the Wii board for vestibular rehabilitation, which used proprioceptive training and visual input (11).

VR is used to rehabilitate children with cochlear implants to improve their spatial hearing skills by incorporating visual information into gamified content (12). Here, visual and auditory information is shared as an example of successful cross-modal sensory interaction.

The use of transcripts has been widely recognized for decades as a support for individuals who are hard of hearing when watching television. Newly software-based automated transcripts have changed the working field of simultaneous translators and led to the development of software and microphone systems, allowing for the differentiation of different speakers (e.g., SPEAKSEE^R^).

The recently developed AR glasses enable the integration of transcription software and its visual presentation during a conversation. Related to the design of the glasses, they have an integrative character and allow specific patients with hearing loss or deafness to see, for the first time, a communicative integration.

This study aims to compare different AR systems as a communicative device in terms of their speech to text capturing abilities, identify their advantages and disadvantages, and discuss potential application groups.

## Materials and methods

**Figure 1:**
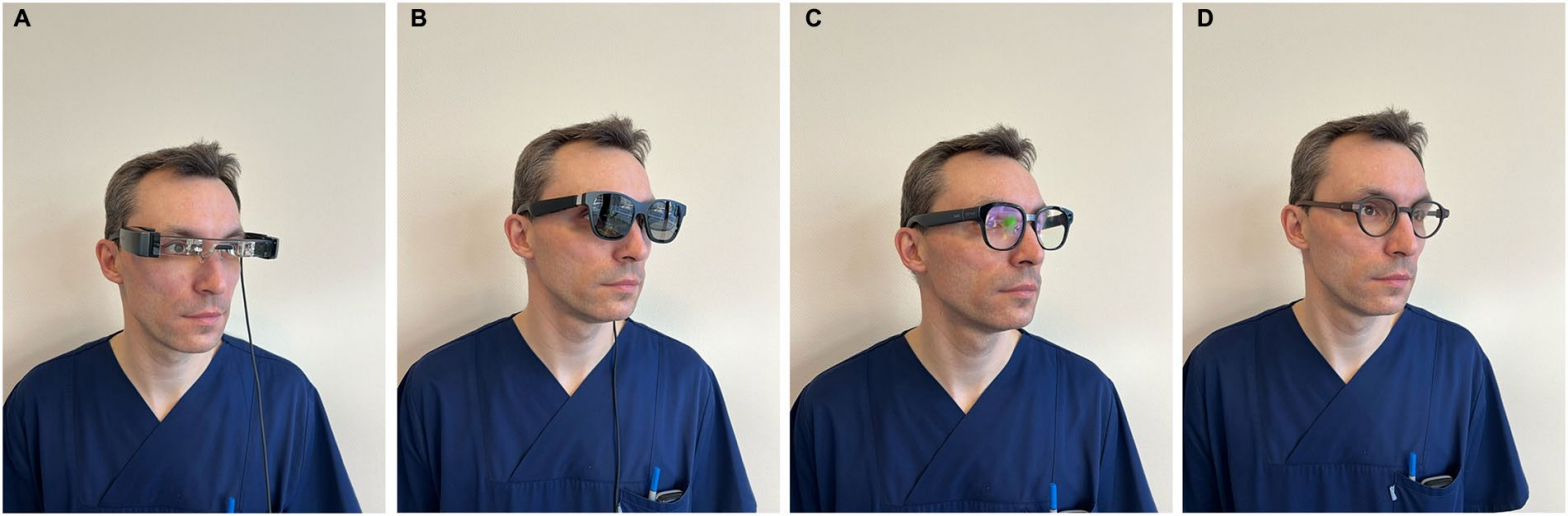
**(A)** Moverio BT 40. **(B)** AIR. **(C)** MYVU. **(D)** G1.

We compared four different systems of AR glasses with the ability for an automated transcript (G1, Even Realities, Shenzen, China; Myvu, Meizu, Guangdong, China; Air, XReal, Haidan, China; Moverio BT 40, Epson, Suwa, Japan) in terms of design, connectivity, software, security design and microphone design. The glasses themselves are shown in Figures 1A–D. The system’s automated transcription capabilities were evaluated in a sound booth using the Freiburger monosyllabic test (at 65 dB and 80 dB), the numbers test (at 65 dB and 80 dB), and the Oldenburger sentence test (OLSA) in an open-set format, both in quiet and in noise conditions. Signal and noise came frontal. The volunteer was normal hearing with regular reading abilities.

**Figure 2:**
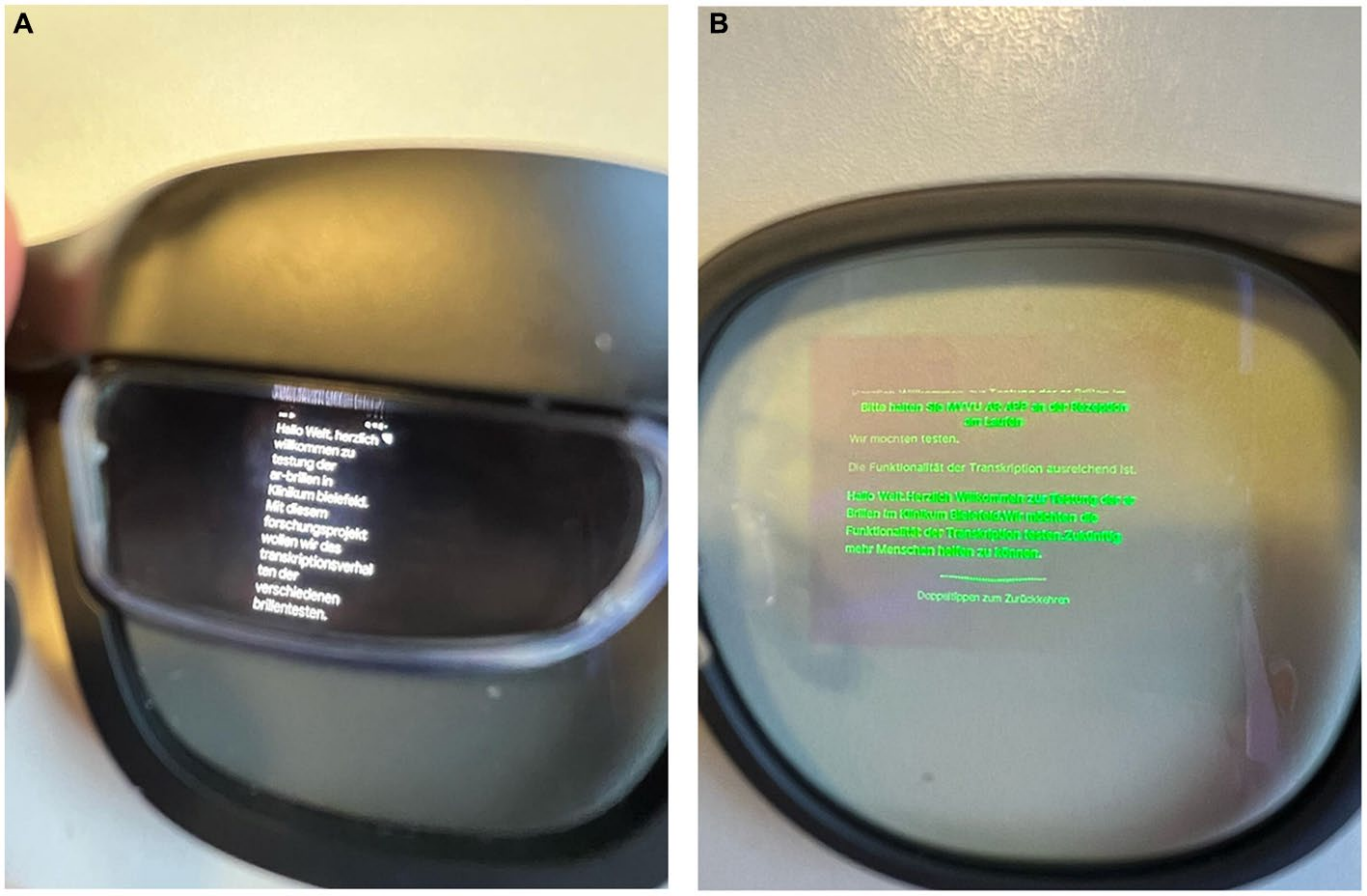
**(A)** View the transcript through AIR glass. **(B)** View the transcript through the MYVU glass.

Besides pure communicative support, additional aspects are essential for evaluating the integrative value of the various systems. Regarding the glass design, the AIR and Moverio are based on a screen principle distinct from the G1 and MYVU glasses. The first design is based on prism glasses, which enable the projection of full-color images across entire browser screens. The software used can be switched from a black letter on a white screen or a white letter to a black screen. In contrast, the latter photodiode projection displays information in letters and numbers in a single color (green) (Figures 2A,B). The G1 system allows for the adaptation of the glasses to the individual visual deficit by adjusting the lens. The AIR system resembles sunglasses, covering the prism glass. Limited system control directly on the glasses is possible for the AIR, G1, and MYVU, either by touching the frame’s sides using the accelerometer through head movements.

Further steering of the systems can be performed by a controller (connected via USB-C) for the AIR and Moverio systems or by Bluetooth and a mobile phone for the G1 and MYVU systems. A direct microphone is integrated into the glasses for the G1, AIR, and MYVU systems. The Moverio has its microphone on the controller. Offline functionality is system-dependent: AIR and Moverio support offline communication by projecting content directly from smartphone or controller screens, while the G1 and MYVU lack this capability entirely.

Implementing Laviere microphones to improve individualization and directionality is possible for the AIR and Moverio systems.

While the G1 and MYVU system-based software allows, besides the speech-to-text transcript, translation into and from different languages, the package contains an AI communicator (ChatGPT, Perplexity for G1; unclear for MYVU), a prompter tool, and a navigation tool. The AIR and Moverio systems have access to the Google apps ecosystem. The Google automated transcript software is regularly used by hearing impaired. The G1 system can be utilized by apps based on AugmentOS (Table 1).

**Table 1:** Comparison of different AR systems.

System	Epson, Moverio 40	Xreal, AIR	Meizo, MYVU IMIKI	Even Realities, G1
Shape/inclusion	−	−	+	+
Display	Prism	Prism	Photodiode	Photodiode
Glasses	Fixed	Fixed	Fixed	Adjustable
Software	Automated transcript, google 6.6.589729414	Automated transcript, google 6.6.589729414	MYVU, 2.32.141	1.5 and access to Augment OS
Microphone	Controller	Glass lateral or controller	Glass lateral	Glass frontal
Connection	USB C	USB C	Bluetooth	Bluetooth
Storage	Controller, EPSON	Controller, XREAL X 4000	Mobile	Mobile
Communication	Offline	Offline	Online	Online

## Results

As a communicative benchmark used in the regular clinical setting, we performed monosyllabic word tests, understanding of numbers, and OLSA testing. This testing allows a comparison with other supportive systems for patients with hearing loss. A primary difference is that speech capturing is stable when using glasses, whereas speech understanding in patients is affected by various variables that influence the auditory pathway.

Patients’ speech understanding of the different systems is shown in Table 2.

**Table 2:** Best speech understanding of different AR systems.

System	Epson, Moverio 40	XREAL, AIR	Meizo, MYVU IMIKI	Even Realities, G1
Numbers 80 dB	100%	100%	95%	100%
65 dB	100%	100%	70%	90%
Monosyllabic 80 dB	75%	65%	55%	80%
65 dB	45%	25%	20%	45%
OLSA in quiet	97%	77%	100%	99%
OLSA in noise	+2.3 dB	+1.7 dB	+ 2 dB	+ 3.5 dB

The tested systems showed good speech-capturing abilities in quiet and for numbers. OLSA’s quiet and tested numbers performance was up to 100%. Speech capturing in noise (OLSA in noise) or in difficult situations (such as monosyllabic speech) was performed for all systems, highlighting the current limitations of the systems.

## Discussion

AR and AI bear enormous potential in many medical fields. In the field of otolaryngology, AI-based transcription software has been used to replace sign language interpreters.

AR glass systems in the gaming industry or industrial use are almost unrelated to communicative content. The combination of transcription software and AR glasses has enormous potential as an additional tool for rehabilitating individuals who are deaf. It allows specific groups to integrate into speech-to-text-based communication for the first time. This means, for example, that people who are dependent on pure sign language-based communication could have access to speech-based communication. A key advantage is the glass’s non-surgical character of informational transmission in cases of severe hearing loss and deafness. Surgery is a limiting point in patients with severe disabilities and comorbidities.

A detailed comparison of the technical abilities of the different systems reveals their deficits, pros, and cons, as well as the specific areas where a substantial improvement could significantly enhance communication or where the difference between the systems is a matter of personal preference. A frontal microphone appears to capture speech more effectively (G1). However, this does not affect hearing in noise in this study.

AIR and Moverio are, in terms of design, less inclusive than MYVU and G1. On- or offline communication is of significant importance in terms of communicative security.

Additionally, it is worth noting that a cross-modal sensory solution, such as AR glasses, is not comparable to a sensory support solution (hearing aids, cochlear implants). We see AR glasses as a solution for patient groups without any other options for inclusion into speech-text-based communication as a supportive tool to improve speech-text-based communication during rehabilitation or as an addition. Clinical cases were a surgery for cochlea implantation even in local anesthesia is not possible are well known (e.g., white cochlea, NF II cases, bilateral traumatic cases). Even in cases were hearing aid use is problematic, AR glasses can be a solution. As support before cochlear implantation or during the rehabilitative process, AR glasses may serve as an informational tool to bridge the gap between acoustic fragments and audiological information. Future clinical experience may reveal additional use cases. Related to the visual integration in communicative rehabilitation, possible influential factors that are currently unclear will become apparent.

In contrast to a sensory support system like hearing aids and cochlear implants, which offer passive support to the patient, the AR system requires active cognitive engagement from hearing-impaired or deaf individuals. This performance is influenced by the ability to read (e.g., alphabetic, age dependence, communicative level, attention, fatigue, …).

The outcome of the speech understanding by the systems depends mainly on the microphone’s ability and the software’s speech recognition quality. It can be assumed that further AI integration of speech-to-noise separation will significantly improve speech capture, similar to what is currently observable in the hearing aid field through AI integration. The potential connection of a Laviere microphone may be crucial in further enhancing transcriptive quality in specific interindividual situations in noisy environments.

The glass design plays a significant role in including deaf persons. Here, regular looks like standard glasses; weight and adjustability are essential for addressing visual impairment. On the other hand, connecting to a controller/ mobile phone limits the mobility of wearing the glasses.

The underlying software, particularly in terms of upgradeability, integration with further applications, and ease of adjustment, is another critical factor. The open-source nature of the software, including Android XR and the Augment OS, presents a potential path for future developments in this direction. This would follow the understanding of seeing AR glasses as a platform for further applications. The limits of the transcripts persist in their inability to convey accentuations that deliver emotions, irony, or cynicism.

A significant drawback of AR systems is the dependence on a second device, such as a mobile phone or a controller, for the transcript. A second device can be lost, and battery lifetime can be a relevant limitation for communication. Another essential aspect is third-party communication, as a mobile online connection or Wi-Fi is dependent on two of the four glass systems. This fact raises concerns about the security of interpersonal communication. Communicative security is essential for widespread use, as the nature of interpersonal communication may not be compromised. We see currently the medical indication for an AR device in two fields: (A) Direct sensory support, if a hearing aid, cochlea implant is not possible. This are, e.g., prelingual deafness and reading ability (sign-language dependence), bilateral nerve deficiency, hearing loss, and chronic otitis externa without the possibility of performing an active middle ear implant surgery. (B) Additionally even for the support for the rehabilitation with hearing aids and CI (e.g., low CI outcome, low HA outcome, temporary use during CI rehabilitation.

The limitations of the systems are evident in their inability to capture directionality. Here, further developments in software and microphone techniques are needed to integrate these crucial points for improved speech capturing. It’s important to underline that is not a clinical study. It compares technically the different devices.

## Conclusion

AR glasses offer a new assistive communication support for select patients with hearing loss in specific indication groups. Current systems have their particular design advantages and disadvantages and should be chosen on an individual base. The systems show limitations in challenging hearing situations.

## Data Availability

The raw data supporting the conclusions of this article will be made available by the authors, without undue reservation.
